# Orally administered yeast‐derived β‐glucan alleviates mast cell‐dependent airway hyperresponsiveness and inflammation in a murine model of asthma

**DOI:** 10.1002/iid3.1333

**Published:** 2024-06-27

**Authors:** Jianzhou Zheng, Yu Bai, Lei Xia, Xiao Sun, Jie Pan, Shizhong Wang, Chunjian Qi

**Affiliations:** ^1^ Laboratory of Oncology The Affiliated Changzhou Second People's Hospital of Nanjing Medical University, Changzhou Medical Center, Basic Research Center Changzhou China; ^2^ Largescale Equipment Platform The Affiliated Changzhou Second People's Hospital of Nanjing Medical University, Changzhou Medical Center Changzhou China

**Keywords:** airway hyperresponsiveness, airway inflammation, asthma, particulate β‐glucans (WGP)

## Abstract

**Background:**

Particulate β‐glucans (WGP) are natural compounds with regulatory roles in various biological processes, including tumorigenesis and inflammatory diseases such as allergic asthma. However, their impact on mast cells (MCs), contributors to airway hyperresponsiveness (AHR) and inflammation in asthma mice, remains unknown.

**Methods:**

C57BL/6 mice underwent repeated OVA sensitization without alum, followed by Ovalbumin (OVA) challenge. Mice received daily oral administration of WGP (OAW) at doses of 50 or 150 mg/kg before sensitization and challenge. We assessed airway function, lung histopathology, and pulmonary inflammatory cell composition in the airways, as well as proinflammatory cytokines and chemokines in the bronchoalveolar lavage fluid (BALF).

**Results:**

The 150 mg/kg OAW treatment mitigated OVA‐induced AHR and airway inflammation, evidenced by reduced airway reactivity to aerosolized methacholine (Mch), diminished inflammatory cell infiltration, and goblet cell hyperplasia in lung tissues. Additionally, OAW hindered the recruitment of inflammatory cells, including MCs and eosinophils, in lung tissues and BALF. OAW treatment attenuated proinflammatory tumor necrosis factor (TNF)‐α and IL‐6 levels in BALF. Notably, OAW significantly downregulated the expression of chemokines CCL3, CCL5, CCL20, CCL22, CXCL9, and CXCL10 in BALF.

**Conclusion:**

These results highlight OAW's robust anti‐inflammatory properties, suggesting potential benefits in treating MC‐dependent AHR and allergic inflammation by influencing inflammatory cell infiltration and regulating proinflammatory cytokines and chemokines in the airways.

## Graphical abstract

Oral administration of WGP (OAW) Treatment Reduces Airway Inflammation and Goblet Cell Hyperplasia 2. OAW Modulates Inflammatory Cell Composition in bronchoalveolar lavage fluid (BALF) and Lungs 3. OAW decreases chemokines and their receptors in the BALF and lung tissues.

## 1 INTRODUCTION

Allergic asthma is a complex respiratory condition characterized by airway hyperresponsiveness (AHR), airway remodeling, and T‐helper cell type 2 (Th2) inflammation. The global increase in asthma prevalence, attributed to factors such as air pollution, occupational exposures, and aero‐allergens, underscores its significance as a public health concern.^1^, ^2^ While inhaled corticosteroids remain the primary treatment for allergic asthma,^3^ their efficacy in reducing eosinophilic airway infiltration may be insufficient for severe asthmatics with mixed granulocytic inflammation,^4^ leading to inadequate responses even at high doses or oral administration.^5^, ^6^ This calls for the development of alternative, safe, and effective therapeutic agents targeting other asthma‐causing effector cells.

Mast cells (MCs) are crucial effector cells in the early phase of allergic reactions, activated by antigen‐mediated cross‐linking of the high affinity IgE receptor (FcεRI). These activated MCs release proinflammatory mediators like histamine and proteases, directly impacting disease severity.^7^ Increased MC numbers in asthmatic airways contribute to variable airflow obstruction and AHR.^8^ While the abnormal accumulation of MCs in the lung is associated with worsening asthma symptoms,^9^ studies suggest that MCs may not be essential for certain features of allergic asthma, including AHR and eosinophil infiltration.^10^, ^11^ However, investigations without adjuvants, such as alum, reveal that MCs may amplify allergic inflammatory responses, particularly in situations of weakened antigen sensitization and challenge.^12^, ^13^

β‐glucans, polysaccharides found naturally in the cell walls of algae, plants, and fungi, exhibit unique antitumour and anti‐infection properties, particularly yeast‐derived particulate β‐glucans (WGP).^14^, ^15^ Recognized by immune cells expressing dectin‐1^16^ and CR3^17^ due to their distinctive β−1,3‐d‐glycoside structure, orally administered WGP (OAW) has demonstrated anti‐infective effects in upper respiratory tract infections^18^ and anti‐inflammatory effects in allergic rhinitis.^19^ In vivo studies have shown the ability of OAW to reduce inflammatory cell infiltration, including eosinophils, in allergic asthma.^20^, ^21^ However, the impact of WGP treatment on the distribution of airway MCs, crucial contributors to AHR and airway inflammation, remains unclear. This study aims to evaluate the efficacy of orally administered yeast‐derived WGP (OAW) on AHR and airway inflammation in a MC‐dependent asthma model.

## 2 MATERIALS AND METHODS

### 2.1 Animals and reagents

Female SPF C57BL/6 mice (6–8 weeks old) were obtained from Changzhou Cavens Company and housed under specific pathogen‐free conditions. Mice were provided with ad libitum access to mouse chow and water. All animal studies followed Traditional Chinese Medical Hospital of Changzhou Animal Care and Use Committee guidelines.

### 2.2 Induction of asthma and OAW administration

**Figure 1:**
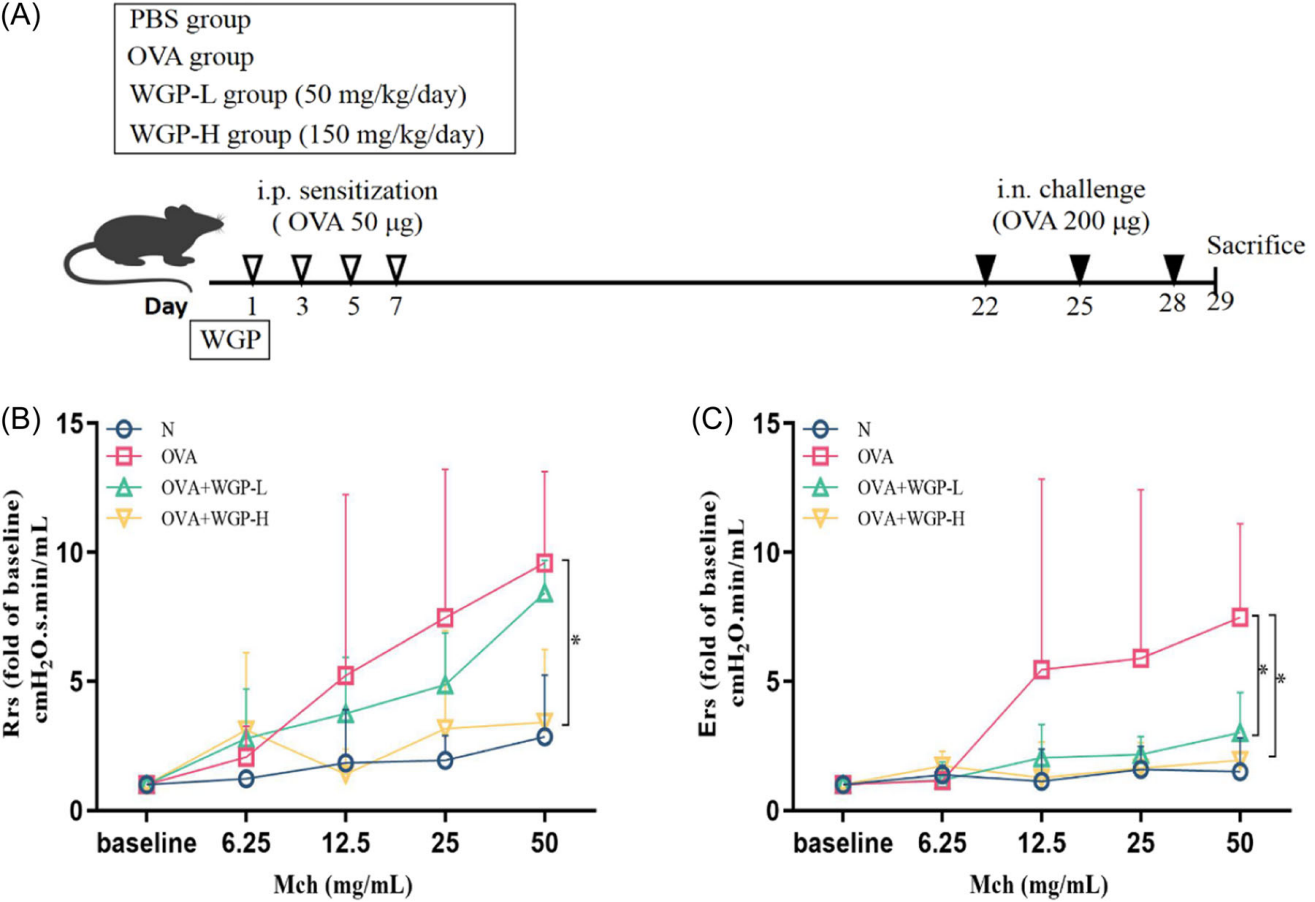
OAW improves airway hyperponsiveness (AHR) in the mast cell‐dependent mouse model of asthma. (A) Schematic representation for OVA‐induced asthma and OAW treatment in C57BL/6 mice. Mice were sensitized with 50 μg ovalbumin (OVA) via intraperitoneal (i.p.) injection on Days 1, 3, 5 and 7, and then challenged with 200 μg OVA by intranasal (i.n.) on Days 22, 25 and 28. Sterile water or WGP (50 mg/kg or 150 mg/kg) in sterile water were administered daily via oral gavage throughout the experiments. On Day 29, there was an analysis of the AHR and collection of BALF and lung tissue for further experiments. Respiratory system resistance (Rrs, B) and respiratory system Elastance (Ers, C) response to increasing dose of Mch were measured 24 h after the last OVA challenge. Lung resistance at PBS (baseline), Mch 6.25, 12.5, 25, and 50 mg/mL. Data are expressed as fold of baseline mean ± SD of each group (*n* = 3/group), *p < .05, compared with OVA‐induced mice using one‐way ANOVA with LSD post‐test. ANOVA, analysis of variance; BALF, bronchoalveolar lavage fluid; Mch, methacholine; OAW, oral administration of WGP.

Mice were randomly assigned to four groups: controls (N), OVA‐induced asthma models (OVA), OAW treatment at 50 mg/kg (OVA + WGP‐L), and OAW treatment at 150 mg/kg (OVA + WGP‐H). The OVA‐induced asthma model involved repeated sensitization and challenge with OVA without alum. OAW treatment (50 or 150 mg/kg) was administered by daily oral gavage starting from OVA sensitization and continuing throughout the experiment, as previously described.^12^ Briefly, Six‐to‐8‐week old C57BL/6 mice were injected intraperitoneally with 100 μL saline or 100 μL saline containing 50 μg OVA on Days 1, 3, 5, and 7. Mice were challenged intranasally with 20 μl saline or 20 μL saline containing 200 μg OVA on Days 22, 25, and 28. On Day 29, the mice were assessed for AHR to methacholine (Mch) (Sigma‐Aldrich) 24 h after the last challenge (Figure 1A). Then, the mice were killed by CO_2_ inhalation, and BALF and lung were collected for subsequent experiments.

WGP isolated from the cell walls of the yeast *Saccharomyces cerevisiae* were provided by Biothera. Mice were treated daily with 100 μL sterile water containing 50 or 150 mg/kg WGP, or with sterile water alone, by oral gavage. Treatment was started OVA sensitization, and was continued for the duration of the experiment (Figure 1A).

### 2.3 Measurement of AHR

To assess the therapeutic efficacy of OAW on pulmonary function in this mouse model, AHR was measured using the FlexiVent system (Flexivent, Scireq) as previously described.^22^ In brief, the experimental mice were intraperitoneally anesthetized with 10% chloral hydrate (Shanghai Zhanyun Chemical Co., Ltd.) and then intubated to the flexiVent ventilator in the supine position. The value of respiratory resistance (Rrs) and respiratory system elastance (Ers), which represent AHR, were evaluated in response to Mch at increasing concentrations (6.25, 12.5, 25 and 50 mg/mL). The airway resistance for each dose of Mch was calculated relative to the baseline saline level.

### 2.4 BALF and lung tissue collection

To assess the therapeutic efficacy of OAW on airway inflammation, BALF was collected after completion of AHR via cannulating the trachea and gently lavaging the airways three times with PBS (total 1 mL, Thermo Fisher Scientific Inc) on Day 29. The collected fluid was centrifuged at 300*g* for 15 min at 4°C to pellet the cells. The supernatant was immediately frozen at −80°C for further analysis. The cell pellets were subsequently resuspended in 200 μL of PBS and used to detect the proportion of inflammatory cells by flow cytometry.

Following lavage, the left lung tissue was removed and embedded in 10% neutral formalin for the preparation of histopathological sections. The right lung tissues were washed twice with ice‐cold PBS. The anterior and middle lobes of the right lung were cut into pieces smaller than 1 mm,^3^ followed by digestion with collagenase type IV, hyaluronidase, and deoxyribonuclease (Sigma‐Aldrich) for 30 min at 37°C on a rotating platform. The samples were then filtered through a 70 μm cell strainer and washed twice with PBS by centrifugation 2000 rpm Finally, a single cell suspension of the lung was obtained for the assessment of inflammatory cell popuations. The lower lobe of the right lung were flash‐freezed in liquid nitrogen and stored in −80°C refrigerator for qRT‐PCR.

### 2.5 Assessment of cytokines and chemotaxis levels

The tumor necrosis factor (TNF)‐α, IL‐6, IL‐10, and IL‐12 levels in the supernatants of BALF were measured using the Mouse ELISA kits (Biolegend, LEGEND MAX™) in accordance with manufacturer's instruction. Levels of 13 mouse chemokines, including CCL2 (MCP‐1), CCL3 (MIP‐1α), CCL4 (MIP‐1β), CCL5 (RANTES), CCL11 (Eotaxin‐1), CCL17 (TARC), CCL20 (MIP‐3α), CCL22 (MDC), CXCL1 (KC), CXCL5 (LIX), CXCL9 (MIG), CXCL10 (IP‐10), CXCL13 (BLC) were measured using Mouse proinflammatory Chemokine Panel (13‐plex) with Filter Plate (Biolegend, LEGENDplex™, NO. 740007) following the manufacturer's instructions. Samples were analysed by flow cytometry on a BD FACS Canto II and data were analysed using LEGENDPlex™ software (Biolegend).

### 2.6 Antibodies and flow cytometry

To assess inflammatory cell infiltration into the airways, the single cell suspension of the lung was blocked with an Fc^−^blocking monoclonal antibody for 15 min on ice, and stained with fluorescently labeled antibodies against antibodies CD45, CD11c, CD11b, Gr‐1, F4/80, CD117, and FcεRIα (Biolegend). The inflammatory cell populations in BALF and lung tissues were measured by flow cytometry as previously described^20^: eosinophils CD45^+^/CD11c^‐^/SiglecF^+^, neutrophils CD45^+^/F4/80^−^/CD11b^+^/Gr‐1^+^, macrophage CD45^+^/F4/80^+^/CD11b^+^/Gr‐1^‐^. MCs were identified by staining lung cells for CD117^+^ FcεRIα^+^. After a washing step, flow cytometry results were acquired using a BD FACS Canto II and analyzed using FlowJo software V8 (Tree Star).

### 2.7 Lung histology

To examine the therapeutic efficacy of OAW on OVA‐induced lung inflammation, the left lung from mice were fixed with 10% neutral formalin for 24 h and then embedded in paraffin. These paraffin tissues were cut into seven micro thick sections. Subsequently, these sections were dewaxed, hydrated and stained with toluidine blue (TB), hematoxylin and eosin (H&E) and periodic acid schiff (PAS) according to standard protocol for histopathological analysis. An Olympus IX‐71 microscope was used to photograph the sections with a bright field microscope. The lung inflammation was assessed using a semi‐quantitative score as previously described^23^: 0, normal; 1, few inflammatory cells; 2, a ring of inflammatory cells 1 cell layer deep; 3, rings of inflammatory cells 2‐4 cells layer deep; 4, rings of inflammatory cells ≥4 cells deep. The lung airway goblet cell hyperplasia was evaluated according to the percentage of PAS positive cells as follows: grade 0, no goblet cells; grade 1, <25%; grade 2, 25‐50%; grade 3, 51‐75%; grade 4, >75%. The number of MCs in airways was quantified in TB staining sections. Histopathology examination was conducted by two pathologists blinded to mouse genotype.

### 2.8 RNA isolation and analysis

Total RNA was extracted from lung tissues using Trizol (Invitrogen, Thermo Fisher Scientific, CN) as the manufacturer's instructions. First‐strand cDNA was synthesized from total RNA with a cDNA Reverse Transcription Kit (Applied Biosystems). Quantitative RT‐PCR was performed using a SYBR Kit (Invitrogen, Thermo Fisher Scientific, CN) as the manufacturer's protocol and normalization with mouse glyceraldehyde 3‐phosphate dehydrogenase (GAPDH). Data were acquired on an ABI ViiA 7 Real‐time PCR system. The primer sequences were designed using online primer 3 free online software. The following primer pairs were used: CCR1, forward primer GTGTTCATCATTGGAGTGGTGG, reverse primer GGTTGAACAGGTAGATGCTGGTC; CCR4, forward primer ACACTGTGACTTCCTCCAGG, reverse primer CACCCTGACTGAAACAAGGC; GAPDH, forward primer TGACCACAGTCCATGCCATC, reverse primer GACGGACACATTGGGGGTAG.

### 2.9 Statistical analysis

All data were expressed as mean ± SD. Significant multiple comparisons between groups were analyzed by one‐way analysis of variance (ANOVA) with post hoc analysis using SPSS 24.0 software. For ANOVA, the homogeneity of variance was firstly tested. If the results were similar, the Least Significant Difference (LSD) 20 test was preceded, and if not, the Dunnett's T3 test was processed. Differences with *p* < .05 was considered as a statistical significance. Statistical diagrams were made by GraphPad Prism 5.0 software.

## 3 RESULTS

### 3.1 OAW treatment improves lung function in the MC‐Dependent asthma model

The efficacy of OAW treatment on lung function was assessed in the MC‐dependent mouse model of asthma. Mice were sensitized with OVA i.p. and challenged with OVA i.n., as previously described.^12^ Subsequently, these mice underwent daily oral administration of low‐dose WGP (50 mg/kg, WGP‐L), high‐dose WGP (150 mg/kg, WGP‐H), or sterile water, as depicted in Figure 1A. AHR was evaluated by measuring respiratory system resistance (Rrs) and elastance (Ers) 24 h after the final OVA challenge, as outlined previously.^22^ Exposure to OVA resulted in a significant increase in Rrs (Figure 1B) and Ers (Figure 1C), indicating heightened AHR at a 50 mg/mL dose of Mch compared to the control group. In contrast, both OAW treatments at 50 mg/kg and 150 mg/kg effectively restored Ers to levels comparable to the response at a 50 mg/mL dose of Mch. While OAW at 50 mg/kg had no significant impact on Rrs, the 150 mg/kg treatment demonstrated an improvement in Rrs in response to 50 mg/mL doses of Mch.

### 3.2 OAW treatment reduces airway inflammation and goblet cell hyperplasia

**Figure 2:**
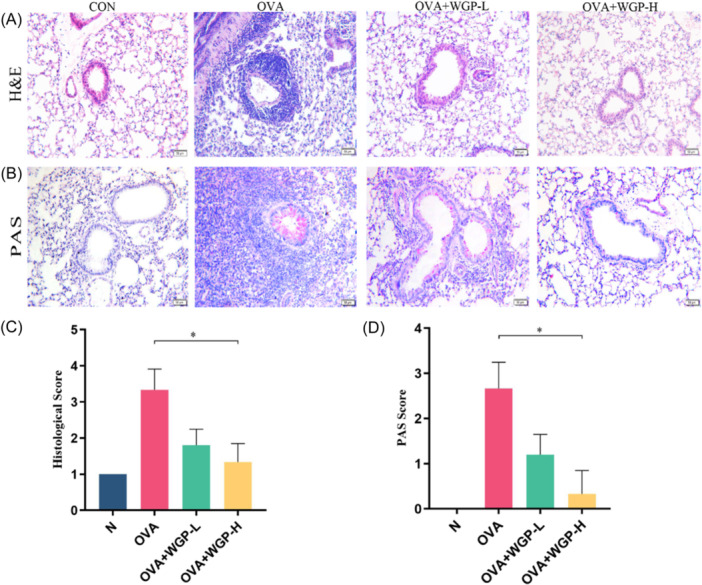
OAW Attenuates Airway Inflammatory Cell Infiltration and Goblet Cell Hyperplasia in OVA‐Sensitized and Challenged Mice (A) Representative hematoxylin and eosin (H&E) stained formalin‐fixed lung sections, illustrating inflammatory cell infiltration. The sections were photographed under light microscopy at ×200 magnification, scale bars represent 50 μm. (B) Periodic acid Schiff (PAS) staining for goblet cells. The sections were photographed under light microscopy at ×200 magnification, scale bars represent 50 μm. (C) Inflammation intensity score, determined by H&E staining as described in Materials and Methods. (D) Quantification of the positive goblet cell area in PAS‐stained sections. Data are presented as mean ± SD of each group (*n* = 3−6/group). *p < .05, compared with OVA‐induced mice using one‐way ANOVA with Dunnett's T3 test post‐test. ANOVA, analysis of variance; BALF, bronchoalveolar lavage fluid; Mch, methacholine; OAW, oral administration of WGP.

To determine whether OAW treatment is effective in OVA‐induced lung inflammation, lung histopathology was performed. The lung sections were stained with H&E and PAS to evaluate airway inflammation after completion of AHR in this study. Consistent with previous studies,^12^, ^24^ we observed a significant increase in inflammatory cell infiltration in the peribronchial areas of OVA‐induced mice, as shown by H&E staining (Figure 2A) and histological scores (Figure 2C), compared to control mice. However, OVA‐induced mice treated with OAW 150 mg/kg exhibited minimal inflammatory cells in the lung sections. Additionally, goblet cell hyperplasia observed in OVA‐induced mice was markedly alleviated with OAW 150 mg/kg treatment, as indicated by PAS staining (Figure 2B) and PAS scores (Figure 2D). It's noteworthy that goblet cells were nearly absent in the lung sections of OVA‐induced mice treated with the 150 mg/kg dose of WGP. Collectively, these findings suggest that OAW at 150 mg/kg effectively attenuates OVA‐induced airway inflammation in the MC‐dependent mouse model of asthma.

### 3.3 OAW modulates inflammatory cell composition in BALF and lungs

**Figure 3:**
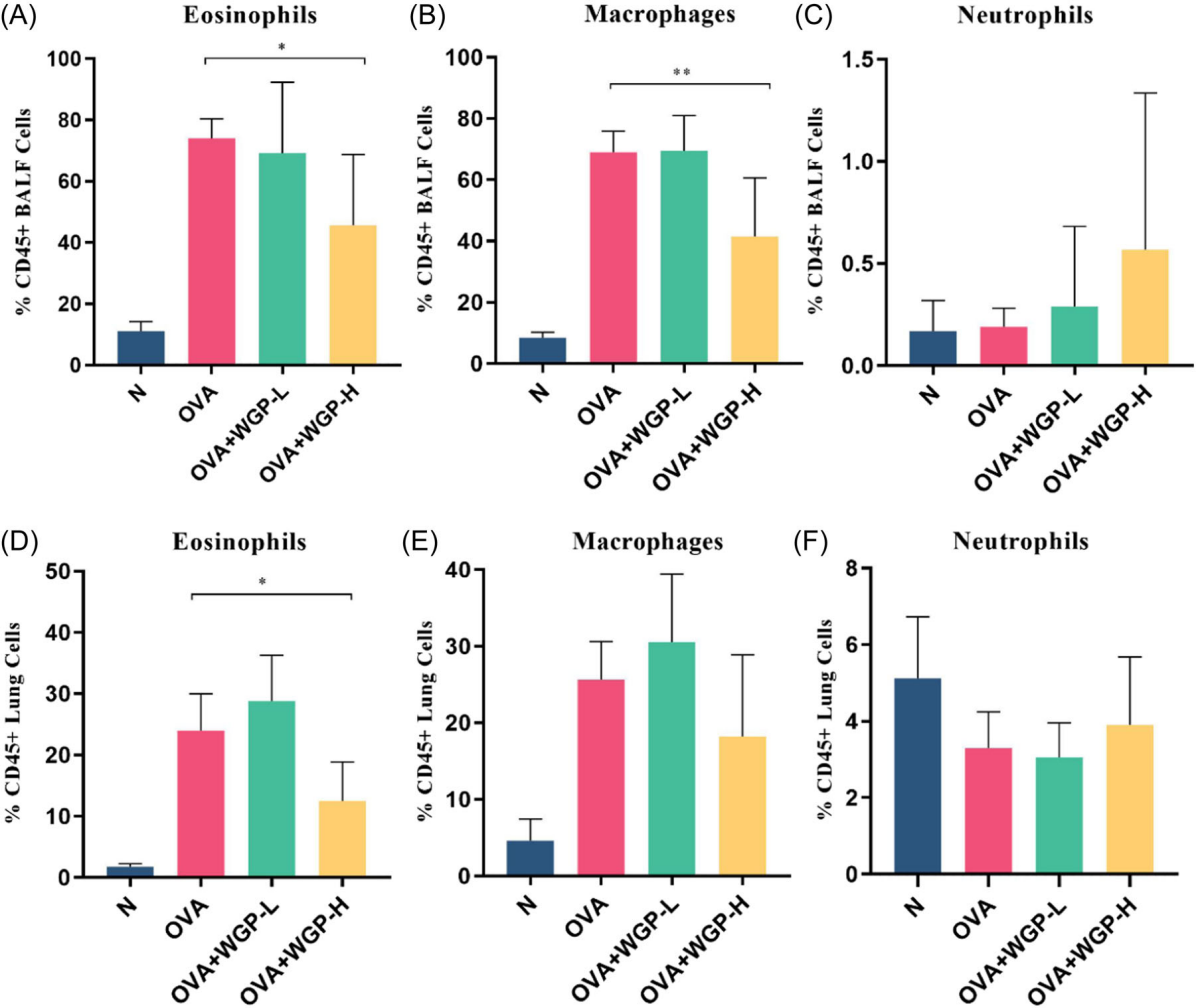
Effect of OAW on the Inflammatory Cell Composition of BALF and Lung in OVA‐Sensitized and Challenged Mice (A−C) Percentages of eosinophils (CD11c^−^/SiglecF^+^), macrophages (F4/80^+^/CD11b^+^/Gr1‐), and neutrophils (F4/80^−^/CD11b^+^/Gr1^+^) in CD45^+^ cells from BALF, determined by flow cytometry. (D−F) Percentages of eosinophils (CD11c^−^/SiglecF^+^), macrophages (F4/80^+^/CD11b^+^/Gr1^−^), and neutrophils (F4/80^−^/CD11b^+^/Gr1^+^) in CD45+ cells from lung tissues, determined by flow cytometry. Data are presented as mean ± SD of each group (*n* = 3−6/group). *p < .05, compared with OVA‐induced mice using one‐way ANOVA with LSD or Dunnett's T3 post‐test. ANOVA, analysis of variance; BALF, bronchoalveolar lavage fluid; Mch, methacholine; OAW, oral administration of WGP.

The OAW treatment modulates the composition of bronchoalveolar lavage fluid (BALF) and pulmonary inflammatory cells. Given the characteristic changes in inflammatory leukocyte infiltrates in asthma, we investigated the impact of OAW on key inflammatory cell types, including eosinophils, macrophages, and neutrophils. Asthmatic mice exhibited a notable accumulation of inflammatory cells, such as eosinophils (CD11c^−^/SiglecF^+^) and macrophages (F4/80^+^/CD11b^+^/Gr1^−^), within CD45^+^ cells in both BALF and lung tissues. In contrast, OAW treatment at 150 mg/kg significantly reduced the percentage of eosinophils in CD45^+^ cells from both BALF and lung tissues of OVA‐induced mice, whereas no significant reduction was observed with OAW at 50 mg/kg (Figures 3A,D; Supporting Information S1: Figure 1). Interestingly, OAW at 150 mg/kg notably decreased the proportion of macrophages in CD45^+^ cells from BALF in OVA‐induced mice, while showing a nonsignificant reduction in CD45^+^ lung tissues (Figures 3B,E; Supporting Information S1: Figure 2). However, there was no significant effect on the percentage of neutrophils (F4/80^−^/CD11b^+^/Gr1^+^) with OAW at either 50 mg/kg or 150 mg/kg (Figures 3C,F; Supporting Information S1: Figure 2). These results suggest that OAW treatment at 150 mg/kg more effectively inhibits the recruitment of airway inflammatory leukocytes, particularly eosinophils and macrophages, to the lungs of these mice.

### 3.4 OAW reduces pulmonary MC recruitment

**Figure 4:**
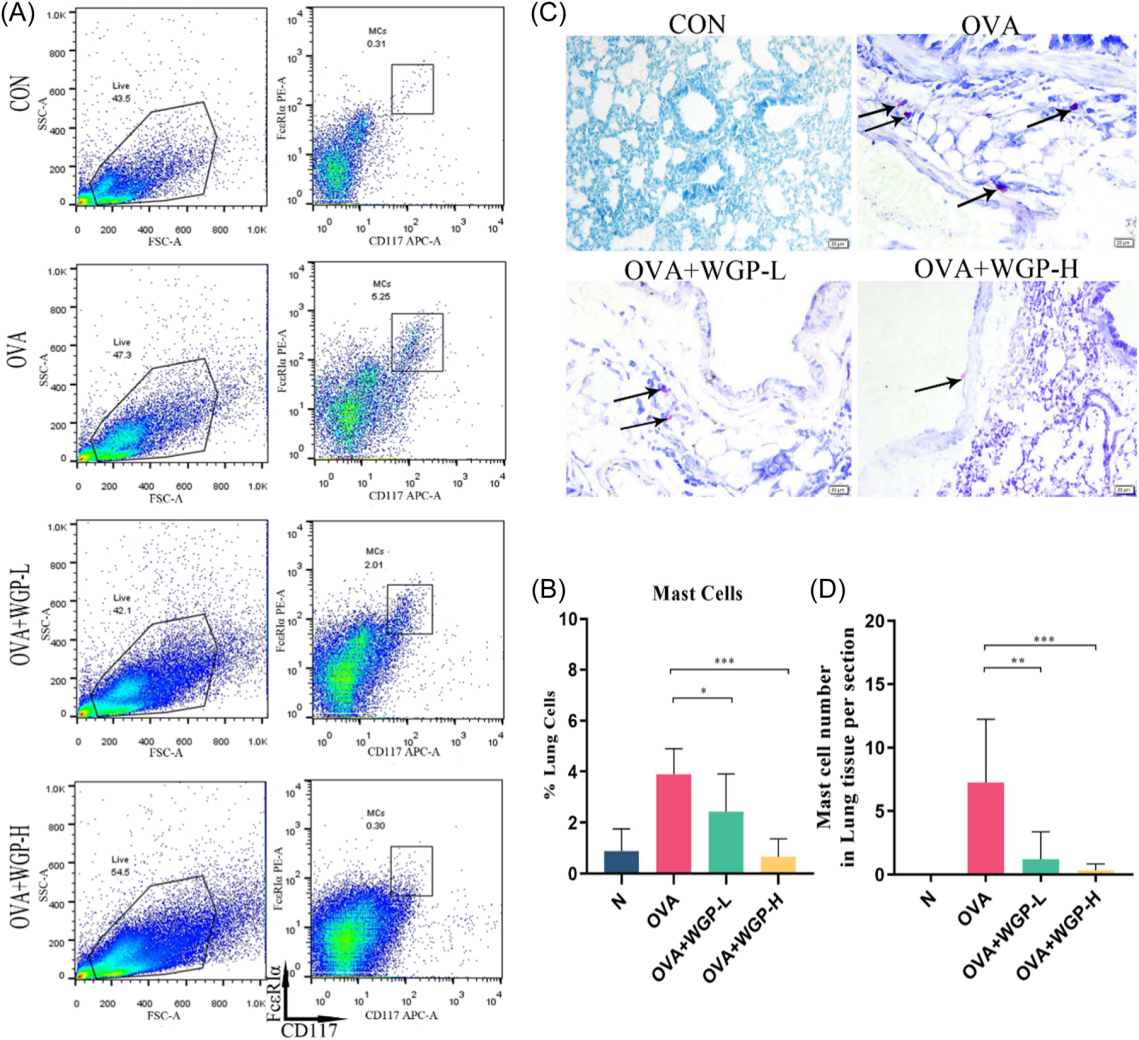
OAW treatment decreases mast cells (MCs) in the lung tissues of mice with OVA Sensitization and Challenge (A) MCs in lung tissues were classified using flow cytometry with characteristic markers FcεRIα+ and CD117^+^. (B) Percentage of MCs quantified. (C) Toluidine blue (TB) stains and (D) the number of MCs in lung sections (indicated with black arrows). The sections were photographed under light microscopy at ×400 magnification, scale bars represent 20 μm. Data are presented as mean ± SD of each group (n = 3‐6/group). *p < .05, **p < .01, ***p < .001, compared with OVA‐induced mice using one‐way ANOVA with LSD post‐test. ANOVA, analysis of variance; BALF, bronchoalveolar lavage fluid; Mch, methacholine; OAW, oral administration of WGP.

Considering the role of MCs in amplifying AHR and inflammation during the early phases of allergic responses, we investigated the impact of OAW treatment on MC recruitment in the lungs. MCs were visualized in lung sections stained with TB and quantified as a percentage of lung cells through flow cytometry. OVA‐induced mice demonstrated an increased recruitment of MCs in lung sections compared to control mice. However, both OAW 50 and 150 mg/kg groups exhibited a significant reduction in the percentage of pulmonary MCs, assessed by flow cytometry for CD117 and FcεRIα staining (CD117^+^ FcεRIα^+^), in comparison to OVA‐induced mice (Figures 4A,B). Furthermore, there was a substantial decrease in the number of MCs in the lungs of OVA‐induced mice treated with low‐dose WGP or high‐dose WGP, compared to the OVA group (Figures 4C,D). Notably, few or no MCs were found in the lungs of OVA‐induced mice treated with OAW 150 mg/kg, indicating the effective inhibition of OAW against pulmonary MC recruitment in the airways, especially at the 150 mg/kg WGP dosage.

### 3.5 OAW decreases chemokines and their receptors in the BALF and lung tissues

**Figure 5:**
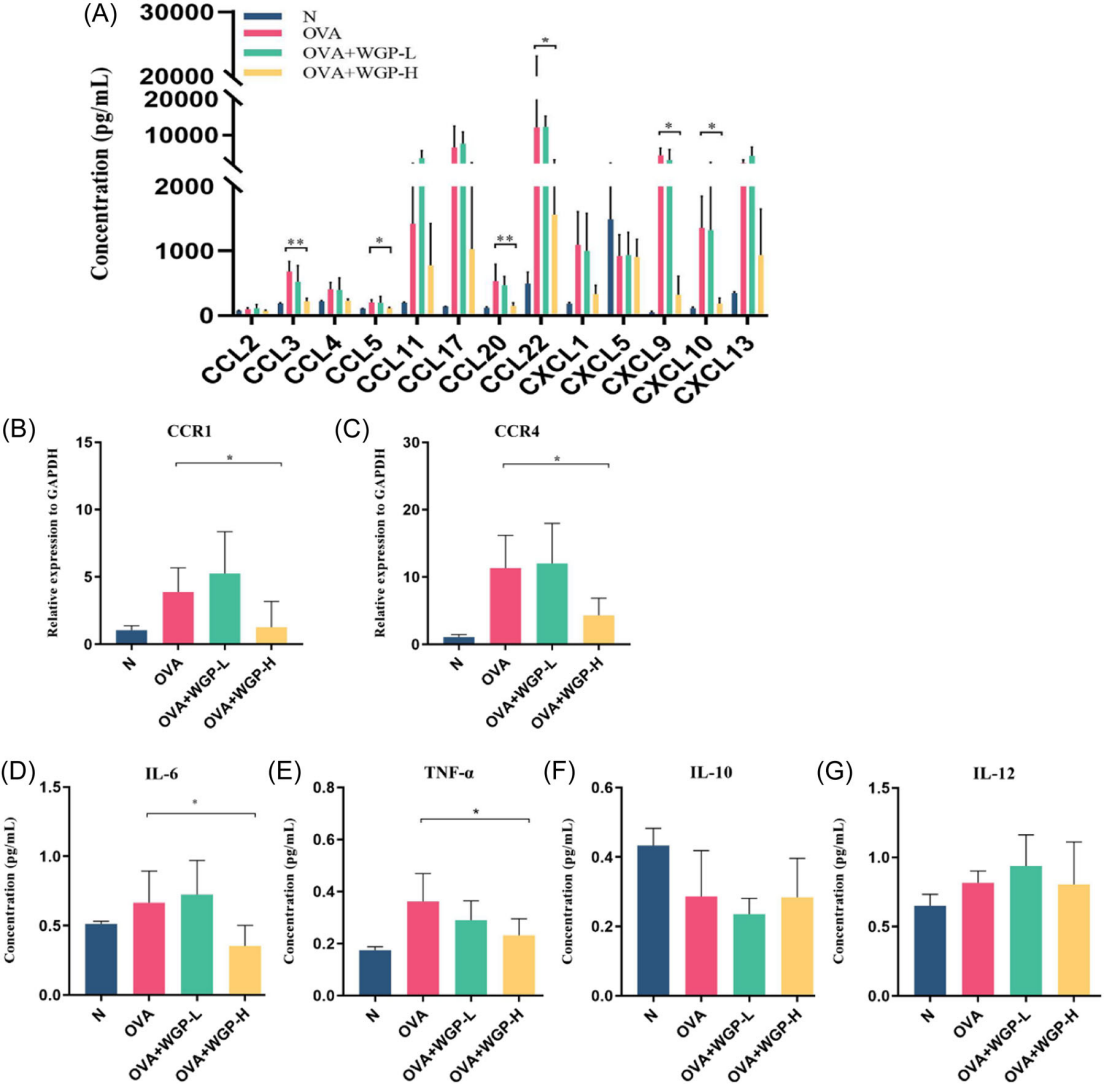
Effect of OAW on Cytokine and Chemokine Levels in BALF and Lung Tissues (A) Concentrations of chemokines in BALF measured by flow cytometry. (B, C) CCR1 and CCR4 mRNA expression levels in lung tissues detected by real‐time PCR. (D−G) Concentrations of cytokines IL6, TNF‐α, IL‐10, and IL‐12 in the BALF from individual mice measured quantitatively by ELISA assay. Data are presented as mean ± SD of each group (*n* = 3−6/group). *p < .05, **p < .01, compared with OVA‐induced mice using one‐way ANOVA with LSD or Dunnett's T3 post‐test. ANOVA, analysis of variance; BALF, bronchoalveolar lavage fluid; Mch, methacholine; OAW, oral administration of WGP.

To understand how OAW influences the recruitment of pulmonary MCs, we examined its impact on chemokine secretion in BALF supernatants using a mouse proinflammatory chemokine assay. In line with previously published data from this model, OVA‐induced mice showed elevated levels of chemokines in the BALF compared to control mice. Conversely, OAW 150 mg/kg treatment remarkably reduced the concentration of multiple chemokines, including CCL3, CCL5, CCL20, CCL22, CXCL9, and CXCL10, in the BALF of OVA‐induced mice (Figure 5A). Furthermore, OAW 150 mg/kg treatment led to a decrease in the mRNA expression levels of chemokine receptors CCR1 and CCR4 (Figure 5B,C), which are receptors for CCL3, CCL5, and CCL22. These reduced concentrations of chemokines and their receptors, known to recruit MCs in asthma,^25^ may explain the inhibition of pulmonary MC accumulation by OAW treatment.

Given the implicated role of Th1/Th2 cytokine imbalance in asthma,^26^ we assessed the levels of IL‐6, TNF‐α, IL‐10, and IL‐12 in the BALF supernatant. IL‐6 and TNF‐α levels were significantly increased in OVA‐induced mice compared to the control group, while IL‐10 and IL‐12 levels remained unaffected (Figure 5D−G). Notably, treatment with OAW at 150 mg/kg significantly reduced IL‐6 and TNF‐α levels in the BALF from asthmatic mice.

## 4 DISCUSSION

The present study aimed to evaluate OAW's impact on AHR and airway inflammation in a MC‐dependent mouse model of allergic asthma. The findings indicated a significant reduction in the inflammatory response in allergic asthma. This was evidenced by a reduction in AHR, airway inflammation, including inflammatory cell infiltration and goblet cell hyperplasia, as well as a decrease in MC infiltration in the lungs. Additionally, the protective effect of OAW was partially attributed to the suppression of the cytokine/chemokine response.

Allergic asthma, a prevalent respiratory disease involving multiple cell types, designates MCs as central players in the asthmatic response due to their release of mediators with airway constrictive and proinflammatory effects.^27^ However, studies using MC‐deficient mice in various asthma models have reported conflicting results regarding the role of MCs in AHR and inflammation.^8^, ^11^, ^12^ Variations within these studies could be attributed to differences in mouse strains, sensitization and challenge types, and the use of allergens with or without adjuvants. In this study, we employed an established MC‐dependent mouse model of allergic asthma, where MCs mainly contribute to AHR and airway inflammation. While OAW has been shown to potentiate immune responses in leukocyte infiltration into inflammation sites,^21^, ^28^, ^29^ its effects on MC infiltration haven't been examined. To our knowledge, this is the first study assessing OAW's effect on asthma features in an MC‐dependent asthmatic mouse model. Notably, OAW significantly alleviated MC infiltration into airways in this asthma model. Moreover, OAW considerably reduced airway reactivity to aerosolized Mch and alleviated airway inflammation symptoms, including goblet cell hyperplasia, eosinophil, and macrophage infiltration in the BALF and/or lung tissues. Thus, we hypothesize that OAW treatment may serve as a therapeutic agent for AHR and airway inflammation development, primarily by reducing MC infiltration into airways. Additionally, the efficacy of OAW in controlling asthma was greater with the potentially optimal dose of 150 mg/kg (WGP‐H) in comparison to 50 mg/kg (WGP‐L).

Proinflammatory cytokines, such as TNF‐α and IL‐6, are known to play a crucial role in various inflammatory diseases. In allergic reactions, MCs can be an essential primary source of TNF‐α.^30^, ^31^ TNF‐α is known to be implicated in the development of AHR and leukocyte recruitment into the lung and airway in asthma.^32^ Notably, extremely low levels of TNF are implicated in the early stages of the chronic inflammatory response.^33^ By contrast, TNF‐/‐ mice sensitized to OVA without alum exhibited significant reductions, including AHR and infiltration of eosinophils, lymphocytes, and neutrophils, compared to wild‐type mice.^34^ Based on its multifaceted proinflammatory properties, mAb to TNF‐α has been developed for asthmatic patient management, demonstrating enhanced lung function, diminished AHR, lower exacerbation frequencies, and enhanced quality of life symptom scores.^35^, ^36^ The level of TNF‐α in BALF increased in OVA‐induced mice but was reversed by treatment with OAW 150 mg/kg. IL‐6, also produced by MCs,^31^ has been found to be elevated in various inflammatory diseases.^37^, ^38^, ^39^ Furthermore, IL‐6 is directly involved in the inflammatory response, promoting leukocyte adhesion and cell migration to tissue^40^ and modulating T cells against apoptosis to increase their survival.^41^ In contrast, blocking the IL‐6 receptor not only decreased eosinophils in the airways but also ameliorated AHR in OVA‐sensitized mice.^42^ In line with these findings, exposure to OVA resulted in a significant rise in IL‐6 levels in BALF, reversed by OAW 150 mg/kg treatment. TNF‐α and IL‐6 may, therefore, be potential targets for AHR and airway inflammation treatment in asthma. Nevertheless, IL‐10 and IL‐12, essential for Th1 cell differentiation, were not inhibited by OAW treatment. Taken together, the AHR and airway inflammation exerted by OAW treatment can partially be explained by the decreased production of proinflammatory cytokines, such as TNF‐α and IL‐6.

C‐C and C‐X‐C chemokines are the two primary groups responsible for AHR and airway inflammation in allergic asthma. It is widely recognized that chemokines and their receptors are involved in recruiting MCs into inflammatory tissues during allergic diseases.^25^ In individuals with allergic asthma, it has been reported that airway MCs contributing to airway remodeling express high levels of CC chemokine receptors CCR1 and CCR4.^43^ Increased expression of CCR1 and CCR4 ligands, including CCL3 (MIP‐1α) and CCL5 (RANTES), in BALF following an allergen challenge.^44^ Moreover, the expression of CCL22 (MDC) for CCR1 and CCR4 ligands was found to be higher in asthma patients infected with rhinovirus. These findings highlight that the presence of CCL3, CCL5, and CCL22 contributes to the homing of CCR1^+^ and CCR4^+^ MCs in asthma.^45^ It is evident that CCL3 and CCL5 also act as intrinsic chemoattractants for MCs expressing CCR5, particularly in asthma.^25^ In agreement with previous studies, we present results demonstrating upregulation of expression of C‐C chemokines CCL3, CCL5, and CCL22 in BALF following OVA challenge. In contrast, these alterations were largely reversed by treatment with 150 mg/kg OAW. Moreover, the chemokine receptor expression of CCR1 and CCR4 in the lungs was significantly reduced by OAW 150 mg/kg treatment.

In addition to C‐C chemokines, C‐X‐C chemokine receptor 3 (CXCR3) may also be a strong candidate for involvement in airway inflammation in asthma. Brightling and colleagues have demonstrated that the highest expression of CXCR3 was found on human lung MCs (HLMC) within the asthmatic airway smooth muscle (ASM).^46^ Secretion of CXCL10 (IP‐10), a ligand of CXCR3, can trigger MC chemotaxis towards ASM. Chemotaxis of HLMC was largely abolished when either CXCR3 or CXCL10 was blocked, indicating that CXCL10 serves as the main chemoattractant for CXCR3+ MCs in the asthmatic ASM. The C‐X‐C chemokine CXCL9 (Mig) and CXCL10 (IP‐10) are CXCR3 ligands.^47^ In agreement with previous studies, we present results demonstrating upregulation of the expression of C‐X‐C chemokines CXCL9 and CXCL10 in BALF following OVA challenge. In contrast, these alterations were reversed by OAW 150 mg/kg treatment. Therefore, we hypothesize that OAW abolishes the transcriptional response of C‐C chemokines and C‐X‐C chemokines, thereby reducing the recruitment of airway MCs.

These cytokines and chemokines play a redundant role in the trafficking of eosinophils and macrophages into the airways, modulating AHR and airway inflammation in asthma.^25^ One limitation of our study is the absence of an experimental group of MC‐deficient mice, which would allow us to determine whether specificity for MCs is present. Nevertheless, the numbers of MCs in OVA‐induced mice treated with OAW were reduced to levels similar to those of normal mice, strongly supporting the fundamental role of OAW treatment in the infiltration of MCs in asthma.

## 5 CONCLUSION

We report that WGP, an active compound derived from yeast, has anti‐inflammatory properties, suggesting potential benefits in treating MC‐dependent AHR and allergic inflammation. The possible mechanism occurs by influencing inflammatory cell infiltration and regulating proinflammatory cytokines and chemokines in the airways.

## AUTHOR CONTRIBUTIONS

The study was conceived and designed by Jianzhou Zheng, Chunjian Qi, and Yu Bai. Jianzhou Zheng: Performed the statistical analysis and Chunjian Qi: Gave advice regarding statistical questions. The manuscript was written by Jianzhou Zheng and Chunjian Qi. Jianzhou Zheng, Chunjian Qi, Yu Bai, Lei Xia, Xiao Sun, and Jie Pan: Contributed to data interpretation and revised the manuscript for important intellectual content. All authors approved the final version of the manuscript.

## CONFLICT OF INTEREST STATEMENT

The authors declare no conflict of interest.

## Supporting information

Supporting information.

## Data Availability

The original contributions presented in the study are included in the article/Supplementary Material, further inquiries can be directed to the corresponding authors.
