# Supplementary material for: Orally administered yeast‐derived β‐glucan alleviates mast cell‐dependent airway hyperresponsiveness and inflammation in a murine model of asthma
Source: Immun Inflamm Dis. 2024 Jun 27;12(6):e1333. doi: 10.1002/iid3.1333 (PMC11209540; doi:10.1002/iid3.1333)
Supplement: Supplementary file 1 — Supporting information. [file IID3-12-e1333-s001.docx]

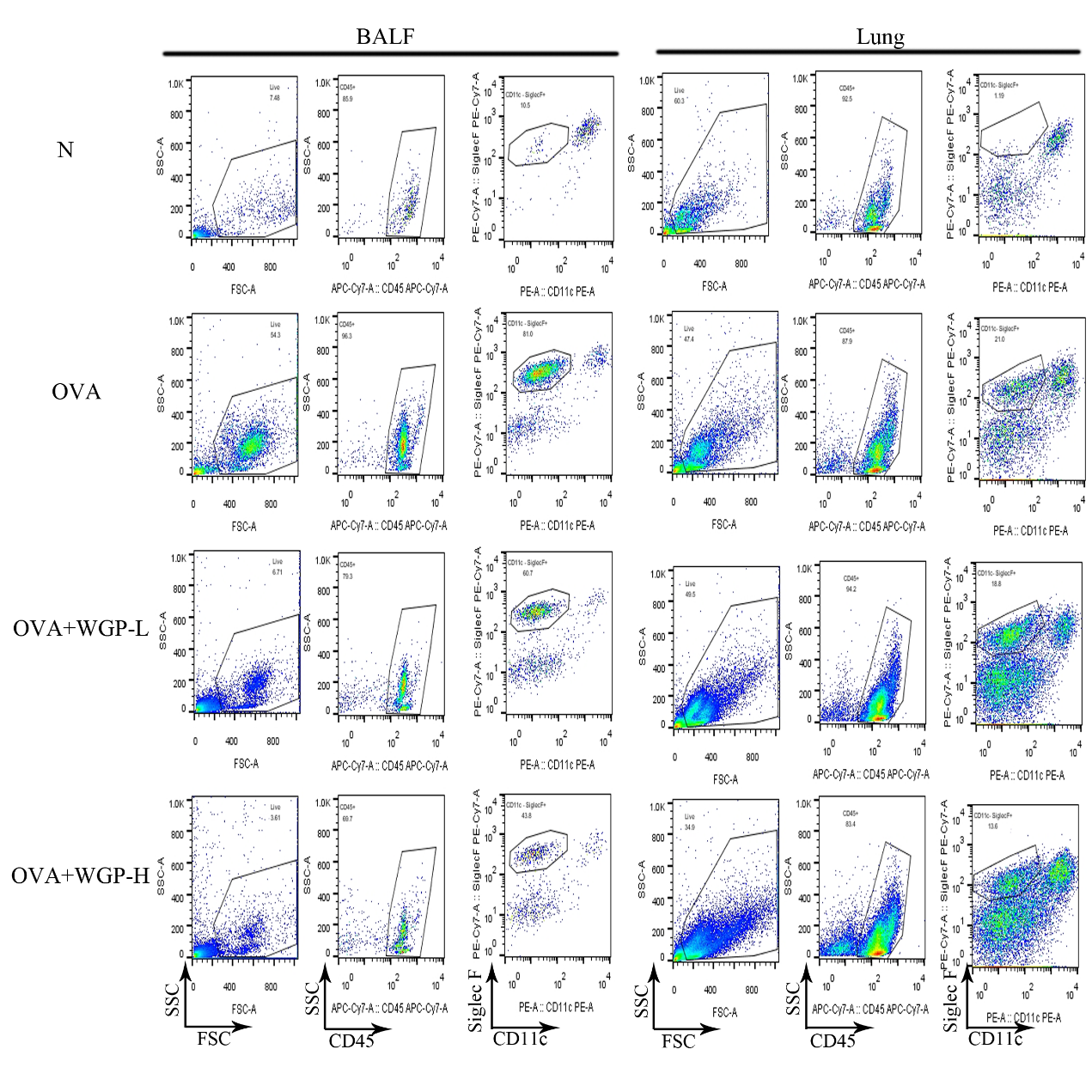


Figure S1. Representative flow cytometry plots of eosinophil percentages in CD45^+^ BALF or CD45^+^ lung cells from OVA-induced mice fed with sterile water, WGP 50mg/kg, or WGP 150mg/kg.


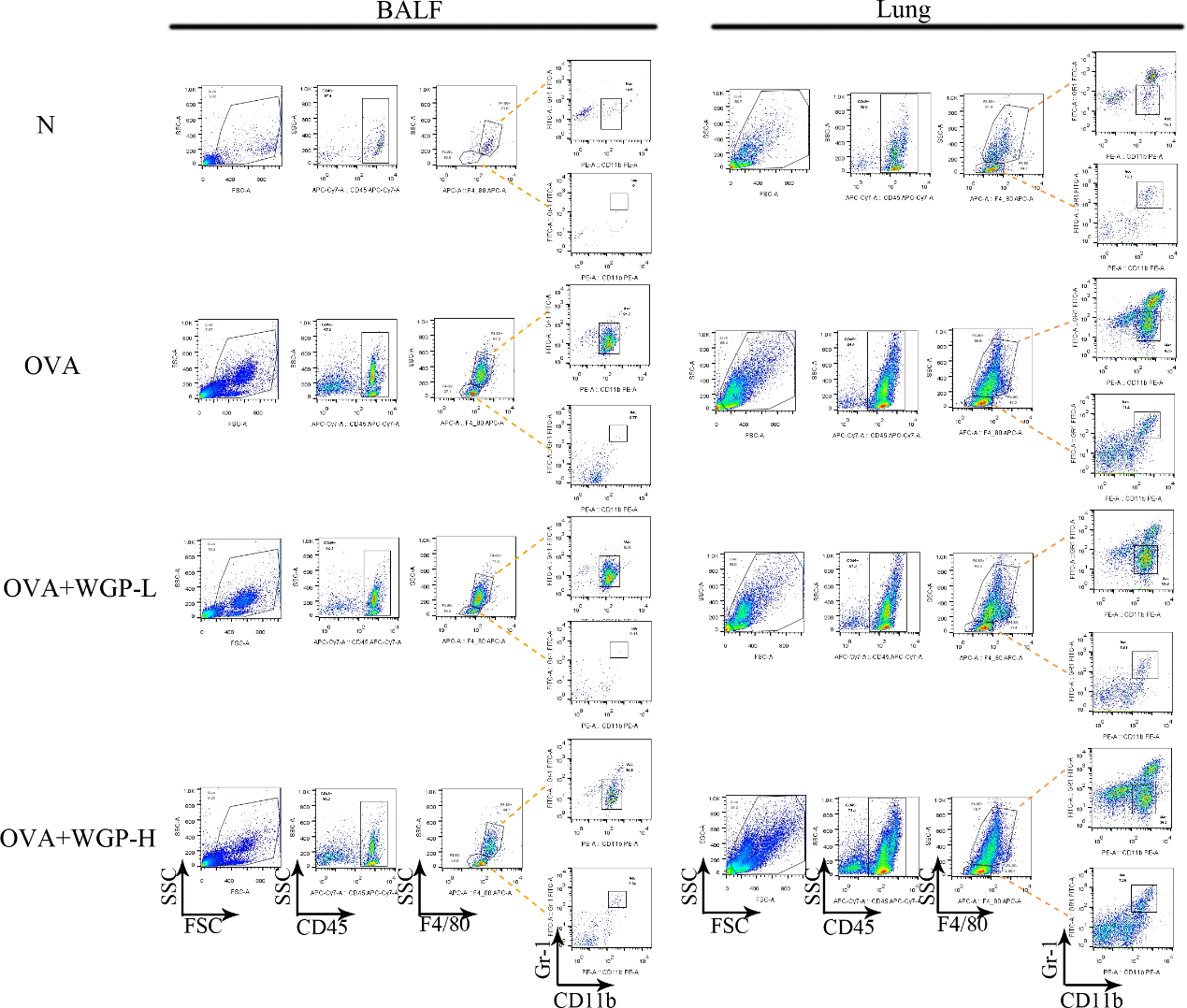


Figure S2. Representative flow cytometry plots of macrophage and neutrophil percentages in CD45^+^ BALF or CD45^+^ lung cells from OVA-induced mice fed with sterile water, WGP 50mg/kg, or WGP 150mg/kg.


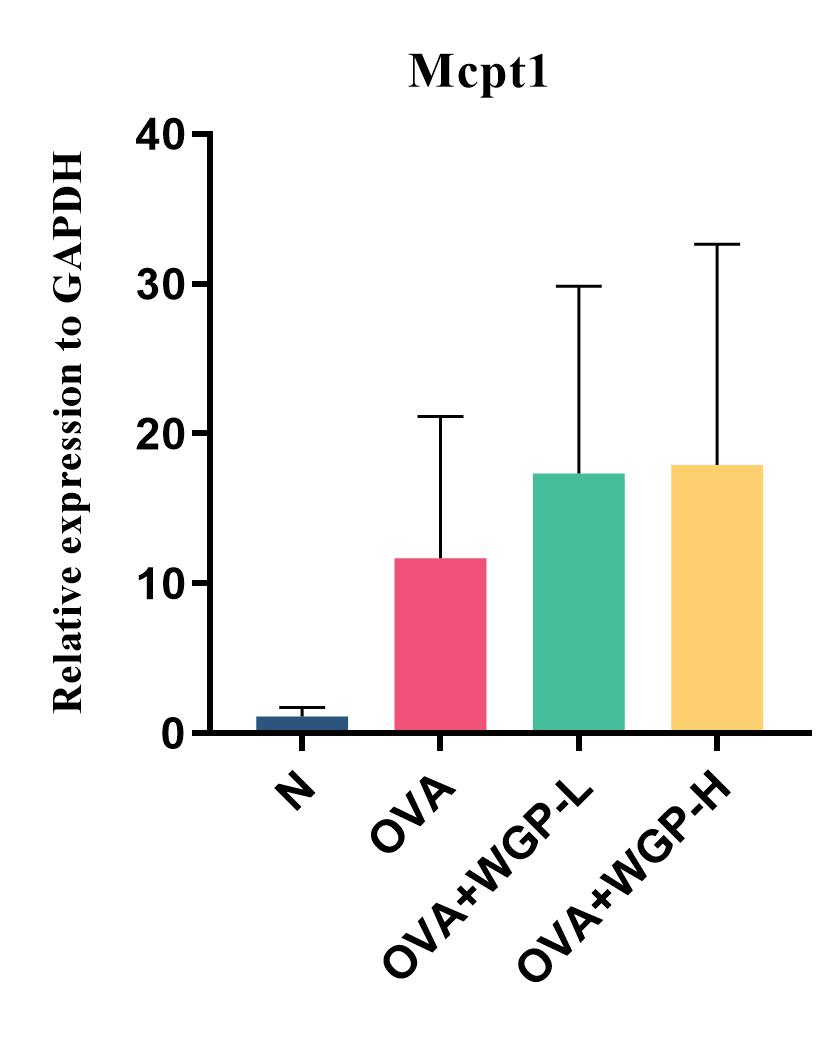

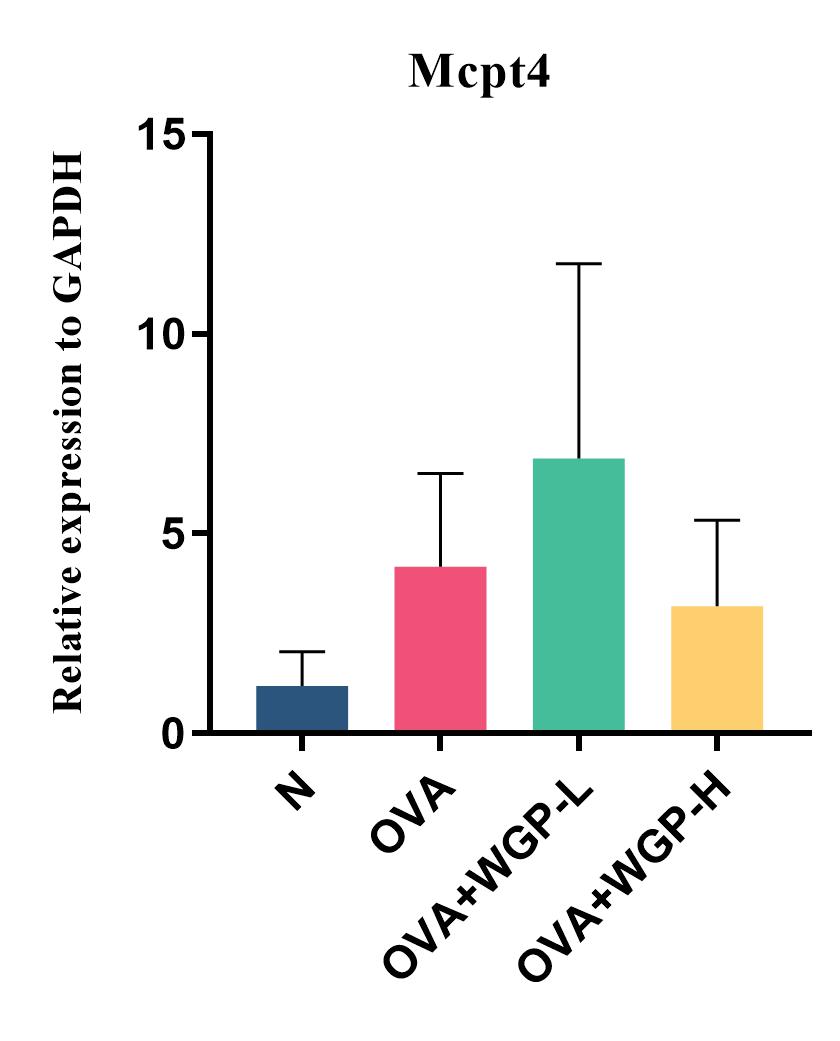


Figure S3. RNA was extracted from lung tissues in mice. Mcpt1 and Mcpt4 mRNA expression levels in lung tissues detected by real-time PCR. Data are presented as mean ± SD of each group (n = 3-6 /group).


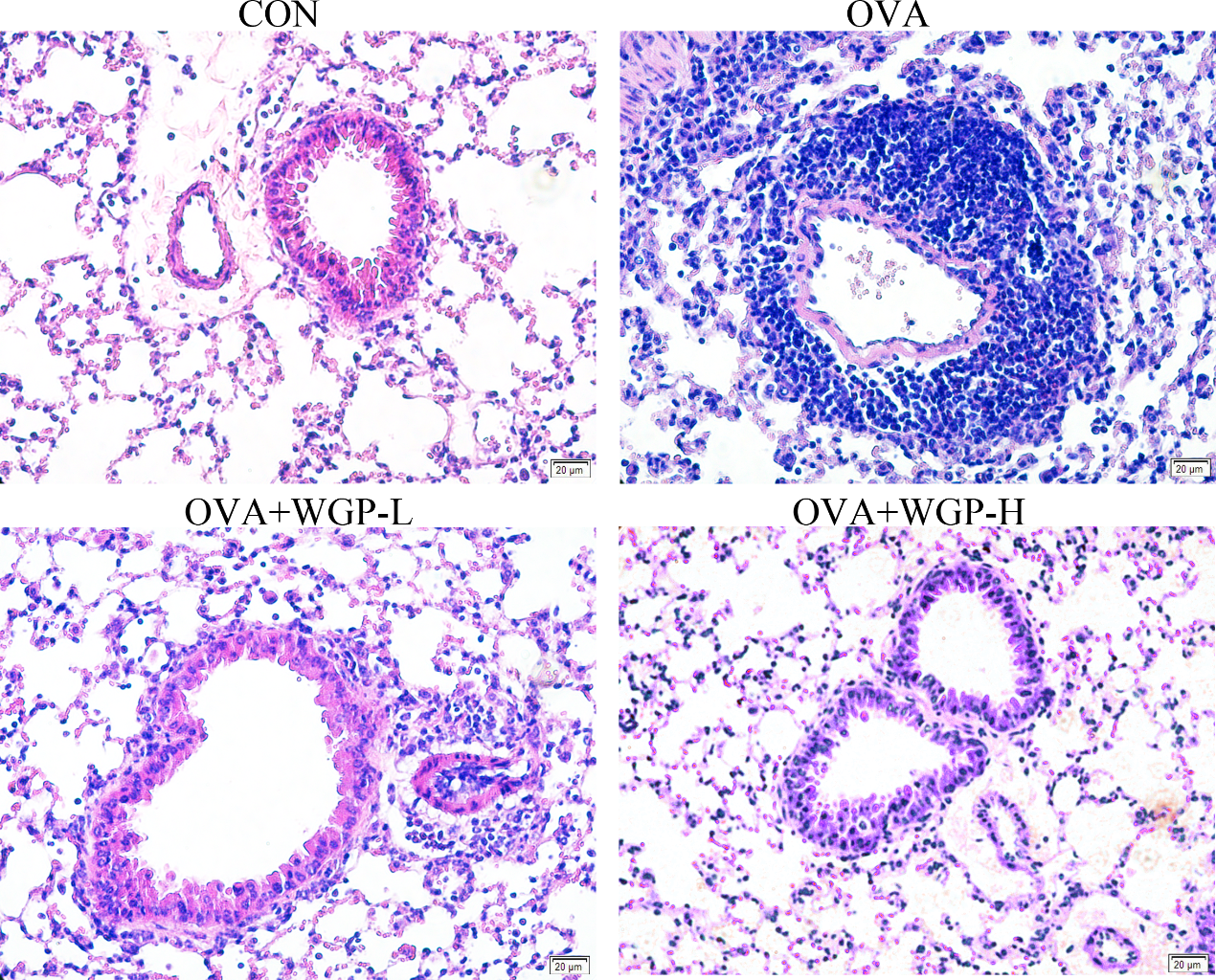


Figure S4. Representative hematoxylin and eosin (H&E) stained formalin-fixed lung sections, illustrating inflammatory cell infiltration. The sections were photographed under light microscopy at ×400 magnification, scale bars represent 20 μm.
